# Bridgework

**Published:** 1897-11

**Authors:** I. N. Carr

**Affiliations:** Durham, N. C.


### Bridgework.

BY DR. I. N. CARR, DURHAM, N. C.

Abstract of a paper read before the Southern Dental Association, Old Point Comfort, Va., August, 1897.

The beauty of all artificial work lies in the ability of the artist to conceal his art, and nowhere is this more desirable than in prosthetic dentistry. In bridgework there are various elements which enter into its proper construction, three of which I desire to mention:

*First.* It must have strength to withstand the stress of mastication.

*Second.* It must be cleanly.

*Third.* It must be artistic in the sense I have mentioned.

Suppose a case requiring the use of a superior cuspid tooth for treatment. To crown the cuspid with gold would be extremely inartistic; to cut it down sufficiently to put on a "window" crown would injure the shape of the tooth, besides showing an unsightly band of gold at the cervical margin. How then shall we secure one end of the bridge to this tooth in the strongest and most artistic manner? Briefly stated I proceed as follows: Grind the palatine surface of the tooth sufficiently to allow a cap of gold to fit over it; then drill three pits, sufficiently deep to cement in three platinum pins, the size of the pins used in artificial teeth, drilling the pits at point most distant from the pulp. Burnish a piece of platinum foil over the surface of the tooth that you have ground down; trim it to shape and thrust the platinum pins through the foil into the pits, allowing them to project sufficiently to come away with the foil when removed, which now proceed to do by means of "sticky wax" softened, and pressed over the foil and pins. Invert in plaster and very fine marble dust; when this has set remove the wax and flow pure gold or 22-k. gold solder over the platinum and pins sufficiently thick to give the necessary strength. With this in position take impression, make articulating model, back up your teeth, put in place and solder. By this method no gold shows except at the tips of the porcelain teeth, and possibly a gold tip on the cuspid. This method was evolved from the fertile brain of our fellow-member Dr. C. C. Alexander. I speak of the advantages of this method of construction in bridgework from personal experience, as I have one in my own mouth, besides having made others to the perfect satisfaction of my patients.

